# Change in Heart Rate Variability after Concussion in a Collegiate Soccer Player

**DOI:** 10.1089/neur.2020.0003

**Published:** 2020-09-29

**Authors:** Forrest L. Anderson, Justin E. Hellwinkel, Marguerite Montjoy, Max Levi, Bin Tu, James M. Noble, Christopher S. Ahmad, Thomas S. Bottiglieri

**Affiliations:** ^1^ Department of Orthopedics, Columbia University Medical Center, New York, New York, USA.; ^2^ Rowan University School of Osteopathic Medicine, Stratford, New Jersey, USA.; ^3^ Columbia Comprehensive Epilepsy Center, Columbia University, New York, New York, USA.; ^4^ Department of Neurology, Taub Institute for Research on Alzheimer Disease and the Aging Brain, and G.H. Sergievsky Center, Columbia University, New York, New York, USA.

**Keywords:** concussion, heart rate variability, return to play, sports medicine

## Abstract

Athletes are known to under-report concussion symptoms due to competitive disincentives to report and conflation of concussion symptoms with other conditions associated with rigorous participation in sports. A quantitative biomarker for concussion has the potential to decrease the reliance on inconsistent patient-reported symptoms for the diagnosis of concussion. The objective of this project was to monitor heart rate variability (HRV) patterns of in-season athletes as a potential biomarker for concussion.

Twenty in-season National Collegiate Athletic Association (NCAA) Division 2 collegiate soccer players were given a wristband heart rate sensor with instructions to wear the band full time (24/7) for the entire fall season (approximately 3 months). The athletes were prompted by email to complete a weekly survey on the severity and frequency of any concussion symptoms. The survey and HRV data were de-identified for confidentiality, and to increase the likelihood of accurate reporting the athletes were told their responses would not be used to disqualify them from athletics. Our hypothesis was that HRV would be diminished in those with recent concussion.

One athlete (5% of the cohort) sustained a concussion during the study period. A marked decrease in HRV was identified 7 days following the concussion, which eventually returned to baseline. This normalization of HRV followed the timing of resolution of concussion symptoms. Participants who did not sustain a concussion exhibited minimal variance in HRV over time.

This preliminary study shows that HRV has potential as a biomarker for symptom resolution after clinically apparent concussion. HRV is unlikely to serve as a concussion diagnostic due to the 7-day lag in HRV change after concussion.

## Introduction

Symptom self-reporting remains the gold standard for concussion diagnosis in contact and collision sports due to the absence of clear, observable signs in the majority of sports-related concussions (SRC).^1^ The need for a reliable, quantitative biomarker of injury occurrence and recovery is apparent. Autonomic nervous system (ANS) dysfunction has been recognized as a marker of post-concussion syndrome.^2^ In recent literature, disruption of heart rate variability (HRV) has been shown to be a measurable aspect of this dysfunction.^3,4^ Although these studies have shown a disruption in HRV,^5^ no study to date has continuously monitored HRV in athletes pre- and post-SRC.

The primary aim of this study was to continuously monitor HR using a non-invasive sensor and derive a daily HRV measurement to identify the timing of ANS dysfunction and recovery post-concussion. We hypothesized that HRV patterns would change in the immediate post-concussion period.

## Methods

### Subjects

Twenty National Collegiate Athletic Association (NCAA) Division 2 collegiate soccer players were given a wristband heart rate sensor with instructions to wear the band full time (24/7) for the entire fall soccer season (approximately 3 months). The athletes selected were listed as starters on the depth chart of the male and female soccer teams. Our medical staff, including team physician and athletic trainer, provided information regarding the study and all 20 starters from each team chose to participate. Twenty devices were available and were offered to position players, but not goalies. Informed consent for participation in this research study was obtained from all participants. The Columbia University Institutional Review Board approved this study.

If concussion was suspected by the athlete, athletic training staff, or coach, the patient was referred for formal clinical evaluation. The diagnosis of concussion was then based on history, physical examination, and special tests including neurocognitive, balance, and reaction time testing. The protocol is included in Supplementary Appendix S1.

### Device study procedures

The WHOOP™ Strap wearable device continuously captures HR via photoplethysmography, which is a validated and accurate method of measuring HR.^6^ A proprietary, patented algorithm is used to derive the final 5 min of rapid eye movement (REM) sleep, during which HR measurements were utilized to calculate HRV using the root mean square of successive differences (RMSSD). The standard deviation of HRV was then calculated for these values over each 24-h period. The device was selected given prior application and acceptance in a college athlete research cohort.^7^

The athletes were also prompted by email to complete a weekly survey on the severity and frequency of any concussion symptoms, alcohol and caffeine consumption, and if any stressful events took place during the week (Table 1). The email and HRV data collected were de-identified for confidentiality and to increase likelihood of accurate reporting and the athletes were told their responses would not be used to disqualify them from athletics.

**Table 1.** Post-Concussion Symptom Scale

Symptoms	Day 1	Day 2	Day 3	Day 4	Day 5	Day 6	Day 7
Headache	1	1	1	0	0	0	0
“Pressure in head”	1	1	1	1	1	1	0
Neck pain	0	0	0	0	0	0	0
Nausea	0	0	0	0	0	0	0
Balance off/Dizziness	0	0	0	0	0	0	0
“Don't feel right”	0	0	1	1	1	0	0
Drowsiness	0	0	0	0	0	0	0
Visual problems	0	0	0	0	0	0	0
Phonophobia	0	0	0	0	0	0	0
Photophobia	0	0	0	0	0	0	0
Confusion	0	0	0	0	0	0	0
Sadness	0	0	0	0	0	0	0
Visual disturbances	0	0	0	0	0	0	0
Feeling slowed down	0	0	1	1	1	0	0
Feeling “dinged”/“dazed”	0	0	0	0	0	0	0
Irritable/Anxious/Nervous	0	0	0	0	0	0	0
Hearing problems/Ringing in ears	0	0	0	0	0	0	0
Feeling tired	0	0	1	1	1	0	0
Feeling like you are “in a fog”	0	0	0	0	0	0	0
Trouble falling asleep	0	0	0	0	0	0	0
Sleeping more than usual	0	0	1	1	1	0	0
Difficulty remembering	0	0	0	0	0	0	0
Difficulty concentrating	0	0	0	0	0	0	0
School/study affected	0	0	0	0	0	0	0
Symptoms with TV/laptop/phone	0	0	0	0	0	0	0
Symptoms during exercise	-	-	-	-	-	-	-
Symptoms after exercise	-	-	-	-	-	-	-
Symptoms the next morning	0	0	1	1	1	0	0
Total symptom score	2	2	7	6	6	1	0

No symptoms = 0, mild = 1, moderate = 2, severe = 3.

### Statistical analysis

Data obtained from the sensor were blinded to participants. All data from the survey were reviewed at the completion of the season. The variability between the daily HRV was plotted for each athlete, and was explored relative to known concussion status. Statistics were calculated using one-way analysis of variance (ANOVA) and the Bonferroni test and were analyzed with GraphPad Prism 8.0 (GraphPad Software, San Diego CA, USA).

## Results

**FIG. 1.**
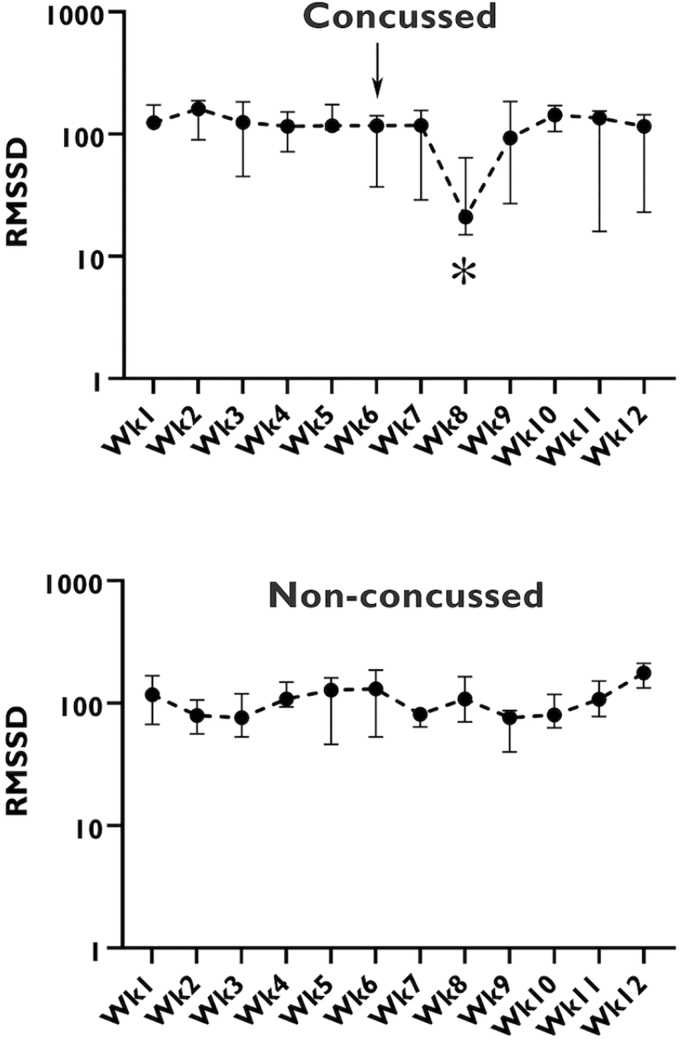
Heart rate variability of two subjects over the study period. The top graph represents data for the study participant who experienced a concussion at the time-point indicated. The bottom graph represents a randomly selected subject who did not experience a concussion during the study period. RMSSD, root mean square of successive differences.

**FIG. 2.**
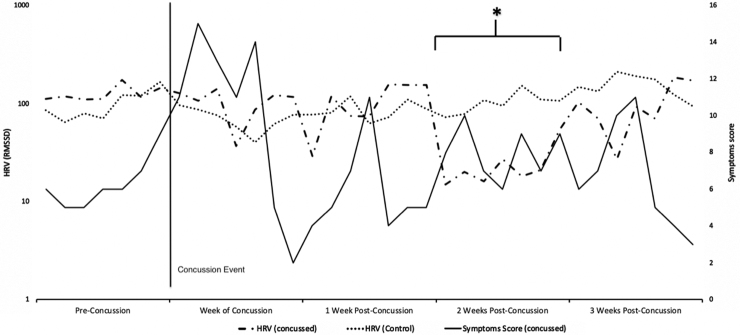
Heart rate variability (HRV) of the concussed subject plotted with that subject's symptom scores and the HRV of a control over the week preceding the concussion event through the week of symptom and HRV normalization, 3 weeks afterwards. HRV between the concussed individual and control was significantly different throughout the second week after concussion. RMSSD, root mean square of successive differences.

Overall, 20 collegiate athletes (10 female [50%], 10 male [50%], age range 18–22 years) completed study procedures, including a full soccer season wearing the WHOOP sensor. During the study period one athlete (5% of the cohort) reported concussion symptoms from a head impact, and was diagnosed with a concussion using the 5th International Concussion in Sport Group (CISG/Berlin 2016) criteria.^8^ The patient was a 19-year-old female. HRV remained at baseline for 7 days following the concussion event, then demonstrated a significant change (*p =* < 0.05) over the following 7 days. The HRV then returned to baseline (Fig. 1). Concussion symptoms correlated well to HRV according to the collected survey data and resolved as the HRV returned to baseline. Figure 2 highlights the week preceding the concussion and 3 weeks following the concussion comparing the symptoms score and HRV of the concussed subject with the HRV of a control. There were no anomalies in the symptoms reported in the anonymous survey by any other study participant that could have represented an unreported concussion.

## Discussion

This pilot study shows that a change in HRV measured using an HR monitor wristband correlated with concussion symptoms after a 7-day delay, which persisted through the resolution of symptoms. The delay in HRV change is unexplained and did not appear to reflect an error in recording. However, HRV did return to baseline as the patient's symptoms resolved.

Although inference is necessarily limited following description of a single case, HRV may nonetheless represent a novel, easily measured non-invasive biomarker for return to play/resolution of concussion symptoms. Considering that athletes often withhold symptom reporting with the aim of minimizing missed time from play, an objective measurement for concussion recovery remains of great interest to the field.^9^ Timing of application and role of any biomarker is important to consider, and can range from informing diagnosis to near- and long-term prognosis. The demonstrated 1 week delay in HRV change after SRC significantly limits the utility of HRV as a biomarker for immediate concussion diagnosis but given its association with symptom resolution, could serve as a non-invasive confirmatory biomarker for concussion recovery and return to sport strategies, potentially in complement with other physiological strategies already in place in the field. Advantages of a wearable physiological measure include objectivity, relatively brief baseline data necessary for within-subject HRV analysis, and application for use over lengthy periods of time, as demonstrated in this study.

Although generalizable conclusions cannot be drawn from this pilot study, our findings are consistent with previous investigations into HRV and autonomic dysfunction in concussed athletes. La Fountaine and colleagues found aberrant sympathetic sino-atrial pacing in a 20-year-old soccer player following concussion.^10^ Mirow and associates also utilized HRV to show autonomic dysfunction in service members after sustaining concussions due to explosions.^11^ However, there are studies that have shown minimal change in HRV in patients after experiencing a mild traumatic brain injury. Su and coworkers found that patients who had sustained a traumatic brain injury and had a Glasgow Coma Scale score of 15 had HRV similar that of controls.^12^

Changes in training/recovery can also impact HRV; however, the HRV changes that occur with athletic training initially follow a depressed measurement for 2–3 weeks with overtraining and then stabilize with continued practice during the season.^13,14^ The depression of HRV with decreased exercise after a concussion is a possibility with deconditioning, but one would not expect the onset of deconditioning to occur so quickly and recover without retraining. Nonetheless, further study of the response of HRV to a reduction in training load, independent of concussion, is necessary to clarify this point.

HRV in this series was measured during sleep each day to limit the impact of artifact during daily activities and exercise. In many studies of HRV, athletes are tested in a clinical setting with protocols directed at controlling activity, movement, and so forth. The proxy of measuring during sleep has been validated and used previously to allow for minimal interruption in patient activity.^11,15^

This study has several limitations. Due to the small sample size, only 1 study participant experienced a concussion during the study period, which limits inference, but suggests a larger study may be warranted. Notably, none of the other 19 athletes demonstrated a rapid or dramatic change in HRV, nor did the concussed athlete in other periods of the season, suggesting a likely valid finding. In addition, the limitation of wearables for detecting sleep states has been studied with other similar products and some have questioned their validity.^15^

## Conclusion

This pilot study demonstrates that HRV could serve as an additional biomarker for concussion resolution, but may not be suitable for confirmation of a concussion diagnosis based on 7-day lag until HRV change following injury in this reported case, and supports further investigation with a larger cohort, improved design, and better measurement tools.

## Supplementary Material

Supplemental data

## Abbreviations Used

ANOVA: one-way analysis of variance
ANS: autonomic nervous system
HRV: heart rate variability
NCAA: National Collegiate Athletic Association
REM: rapid eye movement
RMSSD: root mean square of successive differences
SRC: sports-related concussions
