# Supplementary material for: Change in Heart Rate Variability after Concussion in a Collegiate Soccer Player
Source: Neurotrauma Rep. 2020 Sep 29;1(1):88–92. doi: 10.1089/neur.2020.0003 (PMC8240878; doi:10.1089/neur.2020.0003)
Supplement: Supplemental data [file Supp_Appendix1.pdf]

Supplementary Data

Supplementary Appendix S1. Formal Concussion Evaluation Protocol

1. Concussion history intake:
   a. Injury date
   b. Injury description
   c. Injury location (game, practice, other activity)
   d. When did injury occur (beginning, end)
   e. When did symptoms start
   f. Loss of consciousness
   g. Memory loss
   h. Did you continue to participate with activity despite symptoms?
      i. Any previous concussions? (if YES)
      i. How old when last concussion occurred?
      ii. What sport were you playing?
      iii. Primary symptoms after last concussion
      iv. MOI during last concussion
      v. How long before returning to learn?
      vi. How long before returning to sport?
      vii. Who cleared for return to sport?
      viii. Any other treatments utilized
      ix. Do you wear glasses?
      x. Family history (psychological disorders, migraine headaches, mood or sleep disorder)
      xi. Associated diagnoses (ADD, motion sickness, sleep or learning disorder, anxiety, migraine headaches, dyslexia, depression, anosmia, scalp sensitivity, allodynia)
   j. Emergency room evaluation
   k. Imaging conducted
   l. Team physician evaluation
   m. Athletic trainer evaluation
2. Our physical examination consists of evaluation of cranial nerves, inspecting head and neck for trauma and tenderness, cervical strength and range of motion; Spurling maneuver; static balance assessment; coordination and ocular examination (smooth pursuits, horizontal and vertical vestibular ocular reflex and saccades), convergence testing.
3. In-office testing includes a basic neurocognitive workup utilizing SWAY and Cogstate applications to provide objective information to compare to baseline numbers. These tests offer data for balance, reaction time, impulse control inspection time, delayed recall, memory, and attention.
